# Pros and Cons of Focal Therapy for Localised Prostate Cancer

**DOI:** 10.1155/2011/584784

**Published:** 2011-05-10

**Authors:** Luigi Mearini, Massimo Porena

**Affiliations:** Section of Urology and Andrology, Department of Medical-Surgical Specialties and Public Health, Policlinico Santa Maria della Misericordia Sant'Andrea delle Fratte Perugia, University of Perugia, 06122 Perugia, Italy

## Abstract

In prostate cancer, an interesting and intriguing option to overcome the risks of whole-gland treatment is focal therapy, with the aim of eradicating known cancer foci and reducing collateral damages to the structures essential for maintaining normal urinary and sexual function. Ablation of all known lesions would favorably alter the natural history of the cancer without impacting health-related quality of life and allows for safe retreatment with repeated focal therapy or whole-gland approaches if necessary. Our objective is to reassess the possibilities and criticisms of such procedure: the rationale for focal therapy and the enthusiasm come from the success of conservative approaches in treating other malignancies and in the high incidence of overtreatment introduced by prostate cancer screening programs. One of the challenges in applying such an approach to the treatment of prostate cancer is the multifocal nature of the disease and current difficulties in accurate tumor mapmaking.

## 1. Introduction

Prostate cancer PC remains the most common, noncutaneous male malignancy, with an estimated 186,320 new cases diagnosed in 2008 in the United States [1]. Similar figures are reported in Europe: PC is the most common solid neoplasm, with an incidence of 214 cases per 1000 men [2]. With current trends of PSA testing, and the lowered PSA threshold for biopsy in some Western countries, more and more men will be diagnosed with PC. Although autopsy studies showed that cancer cells can be found in the prostate of 30% to 40% of men at age 60 years (and 60% to 70% at 80 years), the lifetime risk of clinically significant and fatal prostate cancer of a 50-year-old man was estimated to be 9.5% and 2.9%, respectively [3]. 

Recently, the controversy on the benefit of PSA screening, as well as the known side effects of screening itself, served to highlight the concerns about overdetection and consequent overtreatment of patients who fall in the category of the so-called low-risk prostate cancer [4–6]. 

Current treatment of choice for men with localized prostate cancer lies between active surveillance and radical therapy. The rationale of active surveillance for low-risk, low-stage prostate cancer is sound; however, undertreatment is an inherent risk of active surveillance. Nearly one-fourth to one-third of patients, who are thought to be ideal candidates for a policy of no treatment, is later recommended to have therapy once additional information is obtained [7, 8]. In addition, active surveillance, being a “do-nothing” approach, carries the psychological burden of allowing a known cancer to adversely affect quality of life.

Radical prostatectomy is effective; however, because of the risks of postoperative complications (urinary and sexual dysfunction, as great with robotic-assisted prostatectomy as with any other technique [9]), patients with low-risk cancer might be attracted to more conservative alternatives (external beam radiotherapy EBRT or brachytherapy BT). However, they may carry the long-term risks of bowel, sexual, and urinary dysfunction as well [10].

In the last two decades, there has been an awareness of changing treatment paradigms for other cancer: for example, the use of breast sparing surgery, that is, lumpectomy, to treat breast cancer revolutionized the control of that disease: to date, lumpectomy followed by radiation is likely to be equally as effective as mastectomy in selected patients; patient quality of life can successfully be integrated into the equation of cancer treatment without loss of treatment efficacy [11].

Similarly, in urology, small renal lesions may be better suited to partial rather than radical nephrectomy as there is a benefit to maintain as many nephrons as possible. Men with prostate cancer face many of the same issues that breast or kidney cancer patients do, and in recent years the concept of a subtotal therapy has gained the interest of some urological schools [12].

The new goal of future intervention in the treatment of prostate cancer is the Trifecta concept [13]: it means being cured, continent and potent; this is the mainstay of minimally invasive therapy such as focal ablation.

Focal therapy can encompass any degree of subtotal glandular ablation using a variety of devices or techniques derived from experience of whole-gland treatment. Focal therapy is being increasingly discussed as an intriguing treatment option but several issues remain to be addressed.

The aim of this paper is to discuss pro and contra of focal therapy of prostate cancer, presenting possibilities and criticisms according to actual knowledge.

## 2. Materials and Methods

A literature search was done using MEDLINE/Cochrane libraries from 1995 to 2009 using medical subject headings “prostate cancer,” “male lumpectomy,” “focal therapy,” “ablative,” “cryotherapy,” “HIFU,” “laser,” or “photodynamic therapy.”

Original articles, review articles, and editorials published in the years 1995–2009 were included and reviewed in order to select relevant articles.

In November 2010, search for the role of focal therapy or male lumpectomy in prostate cancer produced 492 and 18 references, respectively; searching for prostate cancer, focal therapy, and cryotherapy produced 35 references, of which 21 were reviews; searching for prostate cancer, focal therapy, and HIFU produced 51 references, of which 21 were reviews. Prostate cancer and focal therapy and laser therapy or photodynamic therapy produced 19 and 14 references, respectively (4 and 8 reviews, resp.).

Arguments in favour of or criticisms to focal therapy are discussed and presented together with preliminary experiences.

## 3. Discussion

### 3.1. Pros

#### 3.1.1. Improvement in Detection of Focal Prostate Cancer

In the PSA screened era, prostate cancer as unilateral disease has been shown to exist in 20–40% of men, whilst unifocal disease may be present in up to 70% of men with newly diagnosed localized prostate cancer [14–16]. The Cancer of the Prostate Strategic Urologic Research Endeavor [17] showed that the proportion of patients with unilateral, low-volume tumors with low-risk clinical and pathologic characteristics rose from 29.8% in 1989–1992 to 45.3% in 1999–2001. Polascik et al. [18] showed that among patients with T2 disease, there was a notable increase throughout the years in the proportion of unilateral, low-volume (pT2a) tumors, from 10% in 1988–1995 to 69.4% in 2001–2006. Noguchi et al. [19] demonstrated that the presence of secondary cancers in multifocal prostate tumors did not predict subsequent clinical behavior.

A strong argument against focal therapy is the fact that the majority of men with clinically localized prostate cancer has multifocal disease; however, multifocality included a large proportion of small tumours which may represent clinically insignificant (indolent) disease that are unlikely to progress and impact on quantity of life. 80% of tumour foci has a volume <0.5 cc [20] and may represent inconsequential disease over a 10–15 year period, while all other lesions above 0.5 cc tend to harbour more aggressive disease (higher grade and stage). Furthermore, there are also evidence that there is usually only one clinically significant clone in the prostate being responsible for metastases, and this is the only clinically significant lesion [21]: ablation of the dominant lesion(s) will give rise to disease control [22].

Template transperineal biopsies can serve the purpose to detect the so-called index lesion [23], and it has been accepted as the standard for evaluating patients' eligibility in trials of focal therapy. Crawford et al. [24] reported a 95% accuracy using a perineal brachytherapy template. Similar results were found by Furuno, who found 87% accuracy in comparison with surgical data [25]. Accuracy of prostate mapping with conventional endorectal MRI shows sensitivity up to 85%; the new multiparametric imaging (dynamic contrast-enhanced MRI, diffusion-weighted MRI, and MR spectroscopic imaging [26]) are considered the best imaging for identification and staging of cancer [27–29]. Modern biopsy strategies, combined with optimal imaging and nomograms, provide a strong basis for the inclusion of patients into prospective clinical trials of focal therapy [30].

#### 3.1.2. Avoiding Overtreatment of Low-Risk Prostate Cancer

The European Screening study showed that 1410 men need to be screened and 48 diagnosed in order that one prostate cancer-related death is avoided over a 9-year interval [5]: in this view, some patients are over-treated, and this overtreatment should not be a problem if solution is cost effective and associated with low toxicity. 

The Scandinavian trial [31] comparing surgery versus watchful waiting showed an absolute risk reduction by surgery in preventing cancer mortality within 8 years of 5%, and a recent update showed that this difference did not change at longer followup. However, this difference is probably smaller in a PSA-screened population, since lower-risk disease is detected earlier [32]. The advantage of radical therapy may also become smaller if watchful waiting is substituted with active surveillance which, however, is characterized by a significant dropout [33]. Focal therapy lies between the undertreatment of active surveillance and the overtreatment of radical approaches.

#### 3.1.3. Reduction of Side Effects of Whole Gland Therapy

It is widely demonstrated that radical whole-gland therapy is associated with significant side effects.

Despite improvements in surgical techniques, urinary incontinence UI is not uncommon after radical prostatectomy (RP) [34]: the rate of early UI (3–6 months) varied from 0.8% to 87% and from 5% to 44.5% at 12 months [35, 36]. Several predictors of postoperative UI have been investigated, but unfortunately, they rarely reach a high level of evidence [37]. UI after RP or radiotherapy is caused by sphincter damage [38] and by bladder dysfunction, such as detrusor overactivity and low compliance [39]. UI is a particularly upsetting problem, and it dramatically worsens quality of life QoL [40].

Surgery or radiotherapy causes erectile dysfunction ED in 30–90% of men, with a lower incidence in high-volume center [41]. Despite improvements in surgical techniques, ED is not uncommon after radical prostatectomy [42, 43]: several predictors have been investigated, but unfortunately, they rarely reached a high level of evidence [44]. ED after RP has been related to nerve damage (neuropraxia) resulting in penile hypoxia, smooth muscle apoptosis, fibrosis, and venous-occlusive dysfunction [42], and it is a particularly upsetting problem after RP, worsening quality of life [45]. Patients undergoing radiotherapy develop as well ED in up to 75% of cases, even with the advent of the new protocol of intensity modulated radiation therapy (IMRT) [46].

The best modality to optimize postcancer erectile dysfunction management has not yet been standardized and is still challenging [47–49].

Problems with bowel function rarely occur after RP [50], while Widmark et al. showed that 59% of patients undergoing radiotherapy reported gastrointestinal problems compared with 14% of age-matched controls [51]. In another study, 25% reported moderate and 11% severe bowel changes, including fecal soiling (5%) and bowel frequency (4%) [52]. Another paper has reported greater rates of fecal soiling (10%) and bowel frequency (7%) [53], which can persist for many years. Potosky et al. noted that at 5-year followup, men who had undergone EBRT were still more likely to report bowel urgency and problems with hemorrhoids than men who had undergone surgery [54]. Even brachytherapy has an impact on bowel function: a 3% of rectal bleeding was reported [55], while in a study by Talcott et al., the incidence of rectal urgency was 14% and the incidence of rectal bleeding 6% [56]; Krupski et al. reported that the probability of experiencing problematic diarrhea was 20% at 3 months, 15% at 6 months, and 12% at 9 months [57]. More recently, Miller et al. confirmed that men who underwent EBRT or BT had significantly worse bowel function than men who underwent surgery or age-matched controls [58].

Focal therapy is different from a whole gland treatment: focal means to not target bladder, sphincter, neurovascular bundles, and bowel and should spare continence, erectile function, and overall quality of life.

#### 3.1.4. Improving Overall Quality of Life

All the studies found that changes in quality of life were significantly related to satisfaction with overall outcome among both patients and their partners. Treatment-related changes in quality of life among patients due to a whole gland therapy caused distress in their partners. A multicenter trial showed that a patient's therapy had an effect on the well-being of the patient's spouse or partner [59]: the level of spousal distress arising from a patient's sexual and urinary symptoms after primary prostate-cancer treatment was also associated with the partner's level of satisfaction with the treatment outcome. These findings confirm those of single-institution studies suggesting that patients' urinary or sexual symptoms are problematic for their partners [60].

#### 3.1.5. Cost Saving

Various global economic scenarios are altering the landscape of whole gland therapy. In a randomized trial comparing RP versus watchful waiting WW, at a median followup of 12 years, the overall cost in the RP group was 34% higher than in the WW group [61]. In a recent comparison of open RP versus laparoscopic LRP versus robotic radical prostatectomy RALP, Bolenz et al. showed that RALP is associated with higher cost [62]. Intensity-modulated radiotherapy is more effective but more expensive than conventional radiotherapy [63]. It is possible that in the future health care systems required more cost-effective care finding in focal ablation a cost-effective therapy: effectively, in a comparative study between open RP, LRP, and cryoablation of prostate CAP, the overall direct costs of CAP were offset by the significantly lower nonoperative hospital costs [64].

In conclusion, the overall outcome of whole gland prostate cancer treatment is sensitive to divergent changes in quality of life of patients and their partners due to continence, sexual and bowel function, with increasing costs for the health care system. Investigation on new organ sparing technique [65–70] which could reduce the incidence of adverse events and improve quality of life might represent a valuable new strategy for management of PC.

### 3.2. Cons

#### 3.2.1. Undertreatment of Significant Prostate Cancer

The contra of focal therapy lies in the undertreatment of patients with clinically significant disease. The major arguments against focal therapy can be classified under the broad heading of “understaging” and the understaging argument centers around the multifocality of prostate cancer: multiple foci are found in the majority of specimens [71–73], while it would appear that few patients have true unifocal disease [74]. Low tumor volume is a favorable prognostic feature and predicts low risk of metastasis; however, low tumor volume does not guarantee unifocality. In low-volume disease, there might be multifocal pattern at the same time, even if the size of largest focus “index lesion” seems to determine the patient's prognosis. However, the independent role of Gleason grade cannot be overlooked, and high-grade PC, even in low volume or small foci, represents a risk if untreated.

The concept of treating only the index tumor in selected men is based on a number of pathologic studies. Villers et al. [75] showed that 80% of secondary tumors are <0.5 mL; Rukstalis et al. [76] found that the median size of ancillary lesions was 0.3 cc and proposed that 79% of men would likely have insignificant residual cancer if the index tumor was ablated. However, although treatment of the index tumor has been suggested, to date, there is no technology for reliable disease mapping; moreover, we lack defined followup protocols for the residual prostate that was not ablated and whether the residual prostate would compromise long-term disease control.

#### 3.2.2. Ideal Candidate to Focal Therapy

The major obstacle lies in proper identification of the ideal patient for focal therapy. The most common modality for diagnosing prostate cancer is prostate biopsy, which remains a poor predictor of the extent of tumor on final pathologic specimen: scales found only a 35% positive predictive value of a unilateral biopsy when compared with final RP pathologic specimen [15]; even a saturation biopsy fails to accurately identify the presence of a unilateral tumour in the RP specimen [77]: a mere 22.6% of patients with unilateral core involvement on biopsy had confirmed unilateral PC in the RP specimen, and none of the routinely available clinical and pathological characteristics was found to be an independent predictor of unilateral tumour in the RP specimen.

The consequences of improperly designating a patient for focal therapy may be profound. While the smaller non-index cancer rarely determines prognosis, metastatic deposits have chromosomal alterations discordant with the largest tumor in the prostate, suggesting that nonindex tumors can metastasize [78].

#### 3.2.3. Problems with Diagnostic Tools

Even though template mapping biopsies provide the accuracy needed to administer focal therapy, they have several limitations: there is a higher risk of infection, and in rate of acute urinary retention, extensive sampling increases the morbidity of subsequent RP in patients unsuitable for focal therapy. Saturation biopsy requires anesthesia, and, finally, the cost of the procedure is another important factor to be taken into consideration.

Magnetic resonance imaging has been proposed as an alternative imaging modality to guide prostate biopsy: in a small series including patients with prior negative biopsy, cancer was detected in 55.5% of patients undergoing MRI-guided biopsy [79]; however, the yield of MRI-guided biopsy is similar to repeat systematic TRUS-guided biopsy.

Primary reports for accuracy of endorectal MRI prostate cancer staging show sensitivity up to 85%; however, one of the limitations of MRI is the difficulty of diagnosing central cancer, due to overlapping benign prostatic hyperplasia. Additionally, men who have had recent biopsies often present with hemorrhage, which limits MRI accuracy.

Nevertheless, tumor characterization and risk estimation remain imperfect.

#### 3.2.4. Definition of Cancer Control

The other most problematic area relates to the definition and timing of the surrogate cancer-related outcomes used. Focal therapy will be problematic as some tissue is left untreated, and this inevitably gives rise to PSA increasing. Definitions of biochemical recurrence-free progression are not well established: Lambert et al. [80] use a definition of PSA nadir 50% from baseline after unilateral cryotherapy. Longer followup and validation through other mature series will be needed to define the role of PSA and followup schemes. Absence of cancer in treated lobe is characterized by the same bias of preoperative accuracy of prostate biopsy. The other unanswered question (at least to date) is which retreatment is suitable in case of local persistence or relapse; there are few studies in the literature in the feasibility of radiotherapy, radical prostatectomy, or repeating cryotherapy or HIFU.

#### 3.2.5. Functional Outcomes

Lastly, a presumed but unproven advantage of focal therapy is the lower likelihood and severity of treatment-related morbidity. While this is intuitive, it must be confirmed through trials: treatment-related side effects can be relatively well captured using validated questionnaires in order to compare focal therapy with standard therapies. Questionnaires will be principally directed at genitourinary associated outcomes and at global assessment of quality of life; complication will be classified according the Clavien-Dindo Classification.

## 4. Early Experiences with Focal Therapy

The technique of ablating part of the gland may, in practice, represent the most achievable clinical approach. Focal therapy can be delivered using a number of ablative modalities that treats small, selected tissue, including cryosurgery, high-intensity focused ultrasound HIFU, laser ablation, and photodynamic therapy.

Current strategies for organ-preserving prostate cancer ablative therapy have varied in their eligibility criteria and in the amount of tissue targeted for destruction and/or preservation: nerve-sparing prostate ablation, hemi-ablation, hockey stick, and target focal therapy. Despite paucity of data and a lack of consensus on the most appropriate eligibility and selection criteria, treatment template, and best method of delivering thermal destruction, this approach has become increasingly popular.

### 4.1. Cryosurgery

Preliminary clinical trials of focal cryo-hemiablation suggest that it is feasible, with excellent cancer control and minimal complications although the study design and the scientific standard were poor [80–84]. Focal cryotherapy is a promising option for carefully selected patients although optimization of inclusion criteria is required, since selection criteria are associated with cancer-free survival. Further followup will determine optimal patient selection criteria and followup protocols for patients undergoing primary focal unilateral nerve-sparing prostate cancer treatment. Erectile function is preserved in 68–93% of cases, while no patients reported worsened lower urinary tract symptoms, incontinence, rectal pain, perineal discomfort, or fistula formation [80, 81].

### 4.2. HIFU

HIFU is already being used (unfortunately outside of formal clinical trials), and the preliminary data are encouraging although different eligibility criteria and parameters of treatment, short followup, and absence of patient-reported outcomes make these results hard to interpret.

To date, focal therapy series had evaluated hemiablation of unilateral disease with a Gleason score ≤7, PSA ≤15 ng/mL, clinical stage ≤T2b, using cryosurgery or HIFU. Safety analysis of focal HIFU has demonstrated impotence rates of approximately 15% with little to no incontinence [85, 86].

### 4.3. Laser Ablation

Interstitial laser thermal focal therapy experience is at the beginning, and clinical reports are scarce: in most cases, the literature discusses technical aspects of laser delivery system [87, 88] or Phase I trial [89, 90], reporting interesting results in terms of erectile function sparing and continence, while oncological data are inconsistent for cases and followup.

### 4.4. Photodynamic Therapy

Photodynamic therapy shows promise in delivering focal treatment of both primary and postradiotherapy prostate cancer. Research is ongoing to evaluate mechanism of action of photodynamic energy delivery, development of newer vascular-acting photosensitizers, and potential advantages and disadvantages in focal therapy [91].

## 5. Conclusion

Organ-sparing focal therapy may fill the gap between an active surveillance strategy and whole-gland treatment providing a reasonable balance between cancer control and QoL issues in the future [92].

Focal therapy is still in its infancy. Currently, we do have the optimal basic technology and experience to carry out focal therapy. Candidates for enrollment in trials of focal therapy for localized prostate cancer should meet low-risk criteria based on clinical biopsy and imaging data [79]; with inappropriate patient selection, the potential for missing curative opportunities exists.

The important next steps should be to use an evidence-based approach to study the selection of ideal candidates and subsequently define successful oncologic outcomes of focal therapy. Definitions of success or failure and triggers for re-treatment have not been established.

Early results with cryotherapy and HIFU appear encouraging, even if to date experience is limited and followup is immature.

Feasibility studies for focal therapy of the prostate are underway [93]. Attempting to set and meet guidelines for oncologic efficacy is a challenge we must embrace to safely deliver this revolutionary approach to treating men with prostate cancer.

## References

[1] Jemal A, Siegel R, Ward E (2008). Cancer statistics, 2008. *CA: Cancer Journal for Clinicians*.

[2] Quinn M, Babb P (2002). Patterns and trends in prostate cancer incidence, survival, prevalence and mortality. Part I: international comparisons. *BJU International*.

[3] Whitmore WF (1994). Localised prostatic cancer: management and detection issues. *The Lancet*.

[4] Berg CD, Andriole GL, Crawford ED (2009). Mortality results from a randomized prostate-cancer screening trial. *The New England Journal of Medicine*.

[5] Schröder FH, Hugosson J, Roobol MJ (2009). Screening and prostate-cancer mortality in a randomized european study. *The New England Journal of Medicine*.

[6] Thompson IM, Pauler DK, Goodman PJ (2004). Prevalence of prostate cancer among men with a prostate-specific antigen level ≤4.0 ng per milliliter. *The New England Journal of Medicine*.

[7] Carter HB, Kettermann A, Warlick C (2007). Expectant management of prostate cancer with curative intent: an update of the Johns Hopkins experience. *Journal of Urology*.

[8] Klotz L (2006). Active surveillance with selective delayed intervention for favorable risk prostate cancer. *Urologic Oncology*.

[9] Novara G, Ficarra V, D’Elia C, Secco S, Cavalleri S, Artibani W (2010). Prospective evaluation with standardised criteria for postoperative complications after robotic-assisted laparoscopic radical prostatectomy. *European Urology*.

[10] Pardo Y, Guedea F, Aguiló F (2010). Quality-of-life impact of primary treatments for localized prostate cancer in patients without hormonal treatment. *Journal of Clinical Oncology*.

[11] Santiago RJ, Wu L, Harris E (2004). Fifteen-year results of breast-conserving surgery and definitive irradiation for Stage I and II breast carcinoma: the University of Pennsylvania experience. *International Journal of Radiation Oncology Biology Physics*.

[12] Scardino PT, Abenhaim LL (2008). Focal therapy for prostate cancer: analysis by an international
panel. *Urology*.

[13] Xylinas E, Ploussard G, Durand X (2010). Evaluation of combined oncological and functional outcomes after radical prostatectomy: trifecta rate of achieving continence, potency and cancer control—a literature review. *Urology*.

[14] Djavan B, Susani M, Bursa B, Basharkhah A, Simak R, Marberger M (1999). Predictability and significance of multifocal prostate cancer in the radical prostatectomy specimen. *Techniques in Urology*.

[15] Scales CD, Presti JC, Kane CJ (2007). Predicting unilateral prostate cancer based on biopsy features: implications for focal ablative therapy—results from the SEARCH database. *Journal of Urology*.

[16] Mouraviev V, Mayes JM, Madden JF, Sun L, Polascik TJ (2007). Analysis of laterality and percentage of tumor involvement in 1386 prostatectomized specimens for selection of unilateral focal cryotherapy. *Technology in Cancer Research and Treatment*.

[17] Meiers I, Waters DJ, Bostwick DG (2007). Preoperative prediction of multifocal prostate cancer and application of focal therapy: review 2007. *Urology*.

[18] Polascik TJ, Mayes JM, Sun L, Madden JF, Moul JW, Mouraviev V (2008). Pathologic stage T2a and T2b prostate cancer in the recent prostate-specific antigen era: implications for unilateral ablative therapy. *Prostate*.

[19] Noguchi M, Stamey TA, McNeal JE, Nolley R (2003). Prognostic factors for multifocal prostate cancer in radical prostatectomy specimens: lack of significance of secondary cancers. *Journal of Urology*.

[20] Stamey TA, Freiha FS, McNeal JE, Redwine EA, Whittemore AS, Schmid HP (1993). Localized prostate cancer: relationship of tumor volume to clinical significance for treatment of prostate cancer. *Cancer*.

[21] Liu W, Laitinen S, Khan S (2009). Copy number analysis indicates monoclonal origin of lethal metastatic prostate cancer. *Nature Medicine*.

[22] Ahmed HU (2009). The index lesion and the origin of prostate cancer. *The New England Journal of Medicine*.

[23] Onik G, Barzell W (2008). Transperineal 3D mapping biopsy of the prostate: an essential tool in selecting patients for focal prostate cancer therapy. *Urologic Oncology*.

[24] Crawford ED, Wilson SS, Torkko KC (2005). Clinical staging of prostate cancer: a computer-simulated study of transperineal prostate biopsy. *BJU International*.

[25] Furuno T, Demura T, Kaneta T (2004). Difference of cancer core distribution between first and repeat
biopsy: in patients diagnosed by extensive transperineal
ultrasound guided template prostate biopsy. *Prostate*.

[26] Kurhanewicz J, Vigneron D, Carroll P, Coakley F (2008). Multiparametric magnetic resonance imaging in prostate cancer: present and future. *Current Opinion in Urology*.

[27] Fütterer JJ, Heijmink SWTPJ, Scheenen TWJ (2006). Prostate cancer localization with dynamic contrast-enhanced MR imaging and proton MR spectroscopic imaging. *Radiology*.

[28] Girouin N, Mége-Lechevallier F, Tonina Senes A (2007). Prostate dynamic contrast-enhanced MRI with simple visual diagnostic criteria: is it reasonable?. *European Radiology*.

[29] Yu JS, Chung JJ, Hong SW, Chung BH, Kim JH, Kim KW (2008). Prostate cancer: added value of subtraction dynamic imaging in 3T magnetic resonance imaging with a phased-array body coil. *Yonsei Medical Journal*.

[30] Sartor AO, Hricak H, Wheeler TM (2008). Evaluating localized prostate cancer and identifying candidates for focal therapy. *Urology*.

[31] Bill-Axelson A, Holmberg L, Ruutu M (2005). Radical prostatectomy versus watchful waiting in early prostate cancer. *The New England Journal of Medicine*.

[32] Cooperberg MR, Moul JW, Carroll PR (2005). The changing face of prostate cancer. *Journal of Clinical Oncology*.

[33] van As NJ, Parker CC (2007). Active surveillance with selective radical treatment for localized prostate cancer. *Cancer Journal*.

[34] Touijer K (2007). Urinary continence after radical prostatectomy: “beauty is in the eye of the beholder“. *European Urology*.

[35] van Kampen M, De Weerdt W, van Poppel H, De Ridder D, Feys H, Baert L (2000). Effect of pelvic-floor re-education on duration and degree of incontinence after radical prostatectomy: a randomised controlled trial. *The Lancet*.

[36] Bauer RM, Bastian PJ, Gozzi C, Stief CG (2009). Postprostatectomy Incontinence: all about diagnosis and management. *European Urology*.

[37] Koppie TM, Guillonneau B (2007). Predictors of incontinence after radical prostatectomy: where do we stand?. *European Urology*.

[38] Noguchi M, Shimada A, Nakashima O, Kojiro M, Matsuoka K (2006). Urodynamic evaluation of a suspension technique for rapid recovery of continence after radical retropubic prostatectomy. *International Journal of Urology*.

[39] Stolzenburg JU, Liatsikos EN, Rabenalt R (2006). Nerve sparing endoscopic extraperitoneal radical prostatectomy—effect of puboprostatic ligament preservation on early continence and positive margins. *European Urology*.

[40] Penson DF, McLerran D, Feng Z (2005). 5-Year urinary and sexual outcomes after radical prostatectomy: results from the prostate cancer outcomes study. *Journal of Urology*.

[41] Wilt TJ, MacDonald R, Rutks I, Shamliyan TA, Taylor BC, Kane RL (2008). Systematic review: comparative effectiveness and harms of treatments for clinically localized prostate cancer. *Annals of Internal Medicine*.

[42] Magheli A, Burnett AL (2009). Erectile dysfunction following prostatectomy: prevention and treatment. *Nature Reviews Urology*.

[43] Mulhall JP (2009). Defining and reporting erectile function outcomes after radical prostatectomy: challenges and misconceptions. *Journal of Urology*.

[44] Briganti A, Capitanio U, Chun FKH (2009). Prediction of sexual function after radical prostatectomy. *Cancer*.

[45] Jeffery DD, Tzeng JP, Keefe FJ (2009). Initial report of the cancer patient-reported outcomes measurement information system (PROMIS) sexual function committee: review of sexual function measures and domains used in oncology. *Cancer*.

[46] Brown MW, Brooks JP, Albert PS, Poggi MM (2007). An analysis of erectile function after intensity modulated radiation therapy for localized prostate carcinoma. *Prostate Cancer and Prostatic Diseases*.

[47] Peltier A, van Velthoven R, Roumeguère T (2009). Current management of erectile dysfunction after cancer treatment. *Current Opinion in Oncology*.

[48] Lehrfeld T, Lee DI (2009). The role of vacuum erection devices in penile rehabilitation after radical prostatectomy. *International Journal of Impotence Research*.

[49] Hatzimouratidis K, Burnett AL, Hatzichristou D, McCullough AR, Montorsi F, Mulhall JP (2009). Phosphodiesterase type 5 inhibitors in postprostatectomy erectile dysfunction: a critical analysis of the basic science rationale and clinical application. *European Urology*.

[50] Johnson TK, Gilliland FD, Hoffman RM (2004). Racial/ethnic differences in functional outcomes in the 5 years after diagnosis of localized prostate cancer. *Journal of Clinical Oncology*.

[51] Widmark A, Fransson P, Tavelin B (1994). Self-assessment questionnaire for evaluating urinary and intestinal late side effects after pelvic radiotherapy in patients with prostate cancer compared with an age-matched control population. *Cancer*.

[52] Crook J, Esche B, Futter N (1996). Effect of pelvic radiotherapy for prostate cancer on bowel, bladder, and sexual function: the patient’s perspective. *Urology*.

[53] Franklin C, Parker CA, Morton KM (1998). Late effects of radiation therapy for prostate carcinoma: the patient’s perspective of bladder, bowel and sexual morbidity. *Australasian Radiology*.

[54] Potosky AL, Davis WW, Hoffman RM (2004). Five-year outcomes after prostatectomy or radiotherapy for prostate cancer: the prostate cancer outcomes study. *Journal of the National Cancer Institute*.

[55] Arterbery VE, Frazier A, Dalmia P, Siefer J, Lutz M, Porter A (1997). Quality of life after permanent prostate implant. *Seminars in Surgical Oncology*.

[56] Talcott JA, Clark JA, Stark PC, Mitchell SP (2001). Long-term treatment related complications of brachytherapy for early prostate cancer: a survey of patients previously treated. *Journal of Urology*.

[57] Krupski T, Petroni GR, Bissonette EA, Theodorescu D (2000). Quality-of-life comparison of radical prostatectomy and interstitial brachytherapy in the treatment of clinically localized prostate cancer. *Urology*.

[58] Miller DC, Sanda MG, Dunn RL (2005). Long-term outcomes among localized prostate cancer survivors: health-related quality-of-life changes after radical prostatectomy, external radiation, and brachytherapy. *Journal of Clinical Oncology*.

[59] Harden J, Schafenacker A, Northouse L (2002). Couples’ experiences with prostate cancer: focus group research. *Oncology Nursing Forum*.

[60] Soloway CT, Soloway MS, Kim SS, Kava BR (2005). Sexual, psychological and dyadic qualities of the prostate cancer ‘couple’. *BJU International*.

[61] Andersson S-O, Andrén O, Lyth J (2011). Managing localized prostate cancer by radical prostatectomy or watchful waiting: cost analysis of a randomized trial (SPCG-4). *Scandinavian Journal of Urology and Nephrology*.

[62] Bolenz C, Gupta A, Hotze T (2010). Cost comparison of robotic, laparoscopic, and open radical prostatectomy for prostate cancer. *European Urology*.

[63] Hummel S, Simpson EL, Hemingway P, Stevenson MD, Rees A (2010). Intensity-modulated radiotherapy for the treatment of prostate cancer: a systematic review and economic evaluation. *Health Technology Assessment*.

[64] Mouraviev V, Nosnik I, Sun L (2007). Financial comparative analysis of minimally invasive surgery to open surgery for localized prostate cancer: a single-institution experience. *Urology*.

[65] Ahmed HU, Pendse D, Illing R, Allen C, van der Meulen JHP, Emberton M (2007). Will focal therapy become a standard of care for men with localized prostate cancer?. *Nature Clinical Practice Oncology*.

[66] Eggener SE, Scardino PT, Carroll PR (2007). Focal therapy for localized prostate cancer: a critical appraisal
of rationale and modalities. *Journal of Urology*.

[67] Polascik TJ, Mayes JM, Mouraviev V (2009). Nerve-sparing focal cryoablation of prostate cancer. *Current Opinion in Urology*.

[68] Polascik TJ, Mouraviev V (2009). Focal therapy for prostate cancer is a reasonable treatment option in properly selected patients. *Urology*.

[69] Hou AH, Sullivan KF, Crawford ED (2009). Targeted focal therapy for prostate cancer: a review. *Current Opinion in Urology*.

[70] Ahmed HU, Moore C, Emberton M (2009). Minimally-invasive technologies in uro-oncology: the role of cryotherapy, HIFU and photodynamic therapy in whole gland and focal therapy of localised prostate cancer. *Surgical Oncology*.

[71] Chen ME, Johnston DA, Tang K, Babaian RJ, Troncoso P (2000). Detailed mapping of prostate carcinoma foci: biopsy strategy implications. *Cancer*.

[72] Wise AM, Stamey TA, McNeal JE, Clayton JL (2002). Morphologic and clinical significance of multifocal prostate cancers in radical prostatectomy specimens. *Urology*.

[73] Mai KT, Landry DC, Yazdi HM, Stinson WA, Perkins DG, Morash C (2002). Identification of isolated and early prostatic adenocarcinoma in radical prostatectomy specimens with correlation to biopsy corres: clinical and pathogenetic significance. *Pathology Research and Practice*.

[74] Rice KR, Furusato B, Chen Y, McLeod DG, Sesterhenn IA, Brassell SA (2009). Clinicopathological behavior of single focus prostate
adenocarcinoma. *Journal of Urology*.

[75] Villers A, McNeal JE, Freiha FS, Stamey TA (1992). Multiple cancers in the prostate: morphologic features of clinically recognized versus incidental tumors. *Cancer*.

[76] Rukstalis DB, Goldknopf JL, Crowley EM, Garcia FU (2002). Prostate cryoablation: a scientific rationale for future modifications. *Urology*.

[77] Abdollah F, Scattoni V, Raber M The role of transrectal saturation biopsy in tumour localization: pathological correlation after retropubic radical prostatectomy and implication for focal ablative therapy.

[78] Gburek BM, Kollmorgen TA, Qian J, D’Souza-Gburek SM, Lieber MM, Jenkins RB (1997). Chromosomal anomalies in stage D1 prostate adenocarcinoma primary tumors and lymph node metastases detected by fluorescence in situ hybridization. *Journal of Urology*.

[79] Anastasiadis AG, Lichy MP, Nagele U (2006). MRI-guided biopsy of the prostate increases diagnostic performance in men with elevated or increasing PSA levels after previous negative TRUS biopsies. *European Urology*.

[80] Lambert EH, Bolte K, Masson P, Katz AE (2007). Focal cryosurgery: encouraging health outcomes for unifocal prostate cancer. *Urology*.

[81] Onik G, Vaughan D, Lotenfoe R, Dineen M, Brady J (2008). The “male lumpectomy”: focal therapy for prostate cancer using cryoablation results in 48 patients with at least 2-year follow-up. *Urologic Oncology*.

[82] Ellis DS, Manny TB, Rewcastle JC (2007). Focal cryosurgery followed by penile rehabilitation as primary
treatment for localized prostate cancer: initial results. *Urology*.

[83] Truesdale MD, Cheetham PJ, Hruby GW (2010). An evaluation of patient selection criteria on predicting progression-free survival after primary focal unilateral nerve-sparing cryoablation for prostate cancer: recommendations for follow up. *Cancer Journal*.

[84] Onik G, Narayan P, Vaughan D, Dineen M, Brunelle R (2002). Focal “nerve-sparing” cryosurgery for treatment of primary prostate cancer: a new approach to preserving potency. *Urology*.

[85] Bahn DK, Silverman P, Lee F, Badalament R, Bahn ED, Rewcastle JC (2006). Focal prostate cryoablation: initial results show cancer control and potency preservation. *Journal of Endourology*.

[86] Muto S, Yoshii T, Saito K, Kamiyama Y, Ide H, Horie S (2008). Focal therapy with high-intensity-focused ultrasound in the treatment of localized prostate cancer. *Japanese Journal of Clinical Oncology*.

[87] Atri M, Gertner MR, Haider MA, Weersink RA, Trachtenberg J (2009). Contrast-enhanced ultrasonography for real-time monitoring of interstitial laser thermal therapy in the focal treatment of prostate cancer. *Journal of the Canadian Urological Association*.

[88] Raz O, Haider MA, Davidson SRH (2010). Real-time magnetic resonance imaging-guided focal laser therapy in
patients with low-risk prostate cancer. *European Urology*.

[89] Lindner U, Weersink RA, Haider MA (2009). Image guided photothermal focal therapy for localized prostate cancer: phase I trial. *Journal of Urology*.

[90] Lindner U, Lawrentschuk N, Weersink RA (2010). Focal laser ablation for prostate cancer followed by radical prostatectomy: validation of focal therapy and imaging accuracy. *European Urology*.

[91] Colin P, Estevez J-P, Betrouni N (2011). Photodynamic therapy and prostate cancer. *Progres en Urologie*.

[92] Sanda MG, Dunn RL, Michalski J (2008). Quality of life and satisfaction with outcome among prostate-cancer survivors. *The New England Journal of Medicine*.

[93] De La Rosette J, Ahmed H, Barentsz J (2010). Focal therapy in prostate cancer-report from a consensus panel. *Journal of Endourology*.

